# *In Vitro* Evaluation of the Antioxidant Capacity of 3,3-Disubstituted-3H-benzofuran-2-one Derivatives in a Cellular Model of Neurodegeneration

**DOI:** 10.3390/life14040422

**Published:** 2024-03-22

**Authors:** Sofia Scibetta, Martina Miceli, Marco Iuliano, Luca Stefanuto, Elena Carbone, Paola Piscopo, Vincenzo Petrozza, Giovanna Romeo, Giorgio Mangino, Antonella Calogero, Tecla Gasperi, Paolo Rosa

**Affiliations:** 1 Department of Medical-Surgical Sciences and Biotechnologies, University of Rome Sapienza, Polo Pontino, 04100 Latina, Italy; sofia.scibetta@uniroma1.it (S.S.); marco.iuliano@uniroma1.it (M.I.); vincenzo.petrozza@uniroma1.it (V.P.); giovanna.romeo@uniroma1.it (G.R.); giorgio.mangino@uniroma1.it (G.M.); antonella.calogero@uniroma1.it (A.C.); 2 Department of Science, University of Roma Tre, 00146 Rome, Italy; martina.miceli@icloud.com (M.M.); luca.stefanuto@uniroma3.it (L.S.); 3 Department of Neuroscience, Italian National Institute of Health, 00161 Rome, Italy; elenacarbone96@gmail.com (E.C.); paola.piscopo@iss.it (P.P.); 4 Istituto Chirurgico Ortopedico Traumatologico (ICOT), 04100 Latina, Italy; 5 National Institute of Biostructures and Biosystems (INBB), 00136 Rome, Italy

**Keywords:** neurodegeneration, HO-1, antioxidants, benzofuran-2-ones, oxidative stress, differentiated SH-SY5Y cells

## Abstract

Oxidative stress represents a hallmark for many degenerative pathologies of the Central Nervous System. Throughout life, the constant pressure of noxious stimuli and/or episodes of traumatic events may expose the brain to a microenvironment where the non-balanced reactive oxygen species inevitably lead to neuronal loss and cognitive decline. HO-1, a 32 kDa heat-shock protein catalyzing the degradation of heme into carbon monoxide (CO), iron and biliverdin/bilirubin is considered one of the main antioxidant defense mechanisms playing pivotal roles in neuroprotection. Restoring the redox homeostasis is the goal of many natural or synthetic antioxidant molecules pursuing beneficial effects on brain functions. Here, we investigated the antioxidant capacity of four selected benzofuran-2-one derivatives in a cellular model of neurodegeneration represented by differentiated SH-SY5Y cells exposed to catechol-induced oxidative stress. Our main results highlight how all the molecules have antioxidant properties, especially compound **9**, showing great abilities in reducing intracellular ROS levels and protecting differentiated SH-SY5Y cells from catechol-induced death. This compound above all seems to boost HO-1 mRNA and perinuclear HO-1 protein isoform expression when cells are exposed to the oxidative insult. Our findings open the way to consider benzofuran-2-ones as a novel and promising adjuvant antioxidant strategy for many neurodegenerative disorders.

## 1. Introduction

Oxidative stress (OS) is one of the main leading causes of brain disorders and neurodegenerative diseases such as Alzheimer’s disease (AD) [1], Parkinson’s disease (PD) [2], and amyotrophic lateral sclerosis (ALS) [3]. Both in normal and pathological aging, the imbalance between antioxidant defenses and the accumulation of reactive oxygen species (ROS) chronically exposes the brain to OS, thus initiating the complex process of neurodegeneration and cognitive decline [4]. Either traumatic events such as cerebral ischemia-reperfusion after stroke [5] or exposure to drugs [6], pollutants [7] and radiation [8] are also all sources of OS. The common denominator of the effects of ROS on brain structures is neuronal loss, which is triggered by the oxidation of biological macromolecules (DNA, RNA, proteins, and lipids) [9,10]. According to the mitochondrial hypothesis of neurodegeneration, which asserts that the most susceptible tissues to oxidative phosphorylation system (OXPHOS) flaws are those with the highest energy demand [11], the brain arises as the most vulnerable organ due to its high lipid content (approximately 20% of total body cholesterol) and high dependence on oxygen (almost 20% of total basal oxygen) [12].

Among the many antioxidant defense mechanisms, a pivotal role is played by heme oxygenase-1 (HO-1), an inducible 32 kDa heat-shock protein representing the rate-limiting enzyme for the degradation of heme into carbon monoxide (CO), iron and biliverdin (converted to bilirubin by the bilirubin reductase enzyme) [13]. HO-1 is overexpressed in response to various stress stimuli such as OS, hypoxia, and radiation. Thus, it has predominantly neuroprotective functions unless its induction does not persist, causing the accumulation of the catalyzed reaction degradation products, iron and CO [14]. In the Central Nervous System (CNS), both neuronal and non-neuronal cells upregulate HO-1 in response to cellular stress, with the astrocytes subpopulation being the most able to face the oxidative insults [15]. In AD brains, HO-1 protein is mainly present in neurons, astrocytes, neurofibrillary tangles (NTFs), amyloid plaques, and Cornu Ammonis (CA) of the hippocampus and brain endothelial cells [16]. Moreover, it has been demonstrated that in rat brains HO-1 drives the response to oxidative stress consequently to ischemia-reperfusion injury and that this response is mediated by the nuclear factor erythroid 2-related factor 2 (Nrf2) transcriptional factor [17]. It was also reported that cerebellar granule cells isolated from transgenic mice designed to selectively overexpress HO-1 in neurons show resistance to glutamate and H_2_O_2_-induced oxidative damage [18]. Similarly, a neuroblastoma cell line transfected with a HO-1 expressing construct showed resistance to oxidative stress caused by H_2_O_2_ or β-amyloid_1–40_ [19]. Further, HO-1 is fundamental in brain aging to maintain sterol homeostasis [14,20,21]. To this regard, our group demonstrated that in the aging mouse brain cortex HO-1 expression is controlled by the Early Growth Response-1 (EGR-1) under oxidative stress and regulates the metabolism of brain oxysterols [22].

Based on this, the goal of an ideal therapeutic and/or adjuvant strategy for many brain pathologies should point to restoring redox homeostasis by successfully scavenging ROS or boosting and controlling antioxidant defenses. Several natural or synthetic antioxidants have been not only proposed as adjuvant to conventional pharmacological treatments for neurodegenerative disorders but are also considered as candidate molecules for oxidative damage reduction and are being nowadays used as dietary supplements (the so-called Smart Food) [23,24]. Natural phenolic compounds derived from plants as different forms of vitamin E (tocopherols and tocotrienols) [25], flavonoids (quercetin) [26], gallic acid [27], hydroxytyrosol from olive oil [28], have all shown excellent antioxidant properties with antithrombotic (inhibiting platelet aggregation, endothelial cell activation and LDL oxidation), neuro- and cardioprotective activities [29], which can be explained by the presence of a shared common feature: a catechol functional group [30]. Catechol (C_6_H_4_(OH)_2_), also known as pyrocatechol or 1,2-dihydroxybenzene, is a highly toxic metabolite of benzene, an organic compound extensively used as a volatile solvent in industry or as the starting material for the synthesis of other chemicals [31]. At physiological pH, catechol undergoes auto-oxidation, thus forming quinones and semiquinone radicals, which are more reactive than catechol and play a key role in the generation of ROS, causing DNA and protein damage [32]. Furthermore, catechol induces lipid peroxidation by releasing iron from ferritin [33]. However, compounds with a catechol group reserve outstanding properties by acting as potential therapeutic agents against oxidative stress, inflammatory processes, as neuroprotectors slowing the progression of neurodegenerative diseases and as catechol O-methyltransferase (COMT) inhibitors [34,35]. Currently, such molecules are being employed as stabilizers in cosmetics, industrial preparations and dietary supplements, but result expensive and easily degradable. Hence, there is an increasing need to explore novel synthetic routes as well as to develop novel antioxidants with a broader range of application. Although oxidative stress with its consequences remains among the main targets of the bioactive molecules, the mechanisms by which these compounds exert their beneficial effects are not fully understood.

Prompted by these considerations, this work aimed at evaluating for the first time the antioxidant capacity and the neuroprotective properties of a novel class of compounds synthesized by our group [36] in differentiated SH-SY5Y cells, the most diffused neuronal model for preliminary studies *in vitro* [37]. In particular, we tested four newly synthesized compounds which are characterized by the 3,3-disubstituted-3H-benzofuran-2-one framework decorated with one or more hydroxyl groups on the aromatic ring. The effects of the different compounds were evaluated in differentiated SH-SY5Y cells exposed to catechol stress by assessing cell viability, intracellular ROS generation, and HO-1 expression.

## 2. Materials and Methods

### 2.1. Synthesis of the Benzofuran-2-ones ***6***–***9***

The compounds used for the present work were resynthesized following the protocol already reported in the literature by us [36]. For the full characterization of benzofuran-2-one derivatives **6**–**9**, as well as all for the details of the chemical assays (DPPH, Cyclic Voltammetry), refer to our previous work by Miceli et al. [36].

### 2.2. Cell Cultures and Reagents

The SH-SY5Y human neuroblastoma cell line was purchased from CLS (Cell Lines Service GmbH, Eppelheim, Germany). Cells were grown in Dulbecco’s Modified Eagle Medium (DMEM) supplemented with 10% heat-inactivated Fetal Bovine Serum (FBS, Sigma-Aldrich, St. Louis, MO, USA), 100 IU/mL penicillin G, 100 μg/mL streptomycin, 1% L-glutamine, 1% non-essential amino acids, without sodium pyruvate at 37 °C in 5% CO_2_-humidified atmosphere. At the time of 85% confluence the cells were subcultured, and the medium was changed every 3–4 days. For cell differentiation, SH-SY5Y cells were switched to a DMEM/F12 medium supplemented with 1% FBS in presence of 80 nM phorbol 12-myristate, 13 acetate (PMA, Sigma-Aldrich) for at least 6 days and the actual differentiation was evaluated by the observation of neurites outgrow by microscopy and the evaluation of βIII-tubulin mRNA levels by real-time PCR. Oxidative stress was induced by treating undifferentiated or differentiated SH-SY5Y cells with 10 μM catechol (Sigma-Aldrich). All the synthesized compounds as well as a reference antioxidant, Trolox (TRX, Sigma-Aldrich), were used at a concentration of 10 μM.

### 2.3. Cell Viability Evaluation

#### 2.3.1. Trypan Blue Exclusion Assay

Undifferentiated SH-SY5Y cells (5 × 10^5^ cells/well) were seeded into 6-well plates and maintained overnight. Then, cells were exposed for different times (0–72 h) to increasing concentrations (0–100 μM) of the compounds **6**, **7**, **8**, **9** and TRX. Every 24 h cells were harvested, incorporated with trypan blue and automatically counted by the Countess Cell Counter (Thermo Fisher Scientific, Waltham, MA, USA). Three independent experiments were performed in triplicate, and results were expressed as mean ± SD.

To test catechol toxicity, undifferentiated (1 × 10^6^) and differentiated (1 × 10^5^ at day 0) SH-SY5Y cells were seeded into 35 mm diameter cell culture treated plates and maintained overnight. Then, cells were exposed for 24 h to 10 μM catechol or left untreated, and viability assessed by trypan blue assay and automatically counted by the Countess Cell Counter (Thermo Fishers Scientific). Each experiment was performed three times and results were expressed as mean ± SD.

#### 2.3.2. Cytofluorimetric Analysis of PI Incorporation

Differentiated SH-SY5Y cells (1 × 10^5^ at day 0) were seeded into 35 mm diameter plates and maintained overnight. Then, cells were exposed for 24 h to 10 μM catechol, in presence or not of 10 μM **6**, **7**, **8**, **9** or TRX. Cells were, then, trypsinized, harvested, resuspended in PBS, 2% FBS and incubated with 1 μg/mL propidium iodide (Sigma-Aldrich). The percentage of necrotic cells was evaluated by flow cytometry using a FACs ARIA II instrument (Becton Dickinson, East Rutherford, NJ, USA). At least 2 × 10^5^ events were recorded and analyzed by using FlowJo software v.10.10.0 (Becton Dickinson). Each experiment was performed independently three times.

### 2.4. Cell Proliferation Evaluation

Undifferentiated SH-SY5Y cells (5 × 10^5^ cells/well) were seeded into 6-well plates and maintained overnight. Then, cells were exposed for 72 h to 10 μM catechol and every 24 h the number of cells were incorporated with trypan blue and automatically counted by the Countess Cell Counter (Thermo Fisher). Each experiment was performed independently three times in triplicate and results were expressed as mean ± SD.

### 2.5. Immunofluorescence Analysis

Immunofluorescence analysis was carried out on differentiated and undifferentiated SH-SY5Y cells as previously described, with some modifications [38]. Briefly, undifferentiated SH-SY5Y cells (3 × 10^5^) were plated on glass lenticular in 24-well plates and maintained overnight in DMEM medium supplemented with 10% FBS and treated or not with 10 μM catechol for 24 h (HO-1 staining) or 72 h (Phalloidin staining). For differentiated cells, 2 × 10^4^ SH-SY5Y cells were seeded on glass lenticular in 24-well plates and maintained overnight in DMEM medium supplemented with 10% FBS. Then, cells were subjected to the above-described differentiation protocol (Cell cultures and reagents paragraph). After 6 days of differentiation cells were treated or not with 10 μM **6**, **7**, **8**, **9** or TRX, in presence or not of 10 μM catechol for 6 h. At the end of each experiment, cells were fixed by 4% paraformaldehyde, permeabilized with 0.3% PBS-triton X-100, blocked for 1 h with 10% FBS in PBS and incubated overnight at 4 °C with the following primary antibody: mouse monoclonal anti-HO-1 antibody (sc-136960, Santa Cruz—dilution 1:1000). Goat anti-mouse Alexa-fluor 594 secondary antibody (dilution 1:1000, Life Technologies) was used. For phalloidin staining, cells were directly incubated with Fluoresceine Isothiocyanate-conjugated phalloidin (phalloidin-FITC, P5282, Sigma-Aldrich, dilution 1:100). At the end of the staining procedure, nuclei were counterstained by incubating cells with DAPI for 3 min in the dark. A Nikon Eclipse Ni motorized microscope was used to acquire the images at 40× magnification.

### 2.6. Intracellular ROS Production

The intracellular levels of ROS were determined using dichlorodihydrofluorescein diacetate (DCF-DA, Sigma-Aldrich). Undifferentiated SH-SY5Y cells (1 × 10^6^) were seeded into 35 mm diameter cell culture treated plates and treated or not with 10 μM catechol or 250 μM H_2_O_2_ for 6 h. Differentiated SH-SY5Y cells (1 × 10^5^ at day 0) were seeded into 35 mm diameter cell culture treated plates and treated or not with 10 μM catechol for 6 h, in the presence or not of 10 μM **6**, **7**, **8**, **9** or TRX. At the end of each experiment, cells were stained with 20 μM of DCF-DA for 45 min. 

For fluorescence analysis, qualitative live images were acquired by an inverted fluorescence microscope (Nikon Eclipse Ni) at 20× magnification. 

The fluorescent intensities were also quantified by flow cytometry and expressed as Mean Fluorescence Intensity (MFI). At least 2 × 10^5^ events were recorded and analyzed using Flowing software v2.5.1 (Turku Centre for Biotechnology, University of Turku, Finland). Each experiment was performed independently three times and results are expressed as mean ± SD.

### 2.7. RNA Extraction and Real-Time PCR Analysis

Total RNA was isolated from differentiated and undifferentiated SH-SY5Y cells using Total RNA Purification Kit (Norgen Biotek Corp., Thorold, ON, Canada) as previously described [39]. mRNA concentration was quantified using a Nanodrop spectrophotometer (Thermo Fisher Scientific). One microgram of mRNA was converted to cDNA using the High-Capacity cDNA Reverse Transcription Kit (Applied Biosystem, Warrington, UK) according to the manufacturer’s instructions. Gene expression was quantified by real-time PCR using the ViiA 7 real-time PCR system and Power SYBR Green PCR Master Mix (Applied Biosystem) according to the manufacturer’s instructions. Each experiment was independently repeated three times in triplicate and results were expressed as mean ± SD. Gene expression levels were calculated from real-time PCR data by the method of comparative threshold cycle (CT) using the HPRT1 housekeeping gene as an internal reference. The following gene-specific primers were used: human HO-1: FW 5′-GGGTGATAGAAGAGGCCAAGACT-3′-5′-AGCAACAAAGTGCAAGATTCTGC-3′; human βIII-tubulin: FW: 5′-GCGGATCAGCGTCTACTACA-3′-5′-GGCCTGAAGAGATGTCCAAA-3′; human HPRT1: FW 5′-TGATAGATCCATTCCTATGACTGTAGA-3′-RV 5′-CAAGACATTCTTTCCAGTTAAAGTTG-3′.

### 2.8. Western Blot Analysis

Western blot analysis of differentiated and undifferentiated SH-SY5Y cells total protein extracts was carried out as previously described by our group [40]. Briefly, cell pellets were lysed in Radio ImmunoPrecipitation Assay (RIPA) buffer (50 mM Tris–HCl pH 8.0, 150 mM NaCl, 1% Nonidet P-40, 1 mM EDTA, 0.5% sodium deoxycholate, 0.1% SDS) with protease inhibitors, 1 mM PMSF, 1 mM DTT, and 0.5 mM sodium orthovanadate (Sigma-Aldrich). The Bradford assay (Bio-Rad, Hercules, CA, USA) was used to determine protein concentration. A total of 20 µg of total proteins per sample were resolved on SDS–PAGE gels and blotted onto a PVDF membrane (Amersham HyBond-P GE Healthcare, Chicago, IL, USA). After 1 h blocking at room temperature in 5% dry-milk in Phosphate Buffer Saline (PBS, Sigma-Aldrich) added along with 0.1% Tween-20 (Sigma-Aldrich), membranes were incubated at 4 °C overnight with the following primary antibodies: mouse monoclonal anti-HO-1 antibody (sc-136960, Santa Cruz—dilution 1:1000); mouse monoclonal anti-α-tubulin (T5168, Sigma-Aldrich—dilution 1:10,000) antibody, used as an internal loading control. Membranes were then incubated with anti-mouse horseradish (HRP) peroxidase conjugated secondary antibody (170-6516, Bio-Rad—dilution 1:15,000). Signals were detected by Clarity ECL Western Blotting Substrate (170-5060, Bio-Rad). Digital images were acquired by a ChemiDoc XRS C System (Bio-Rad). Bands intensities were quantified by densitometric analysis using Image Lab software v.6.0.0 (Bio-Rad), and the relative adjusted volumes values were normalized to those of α-tubulin. Each experiment was performed independently three times and results were expressed as mean ± SD.

### 2.9. Statistical Analysis

All statistical analyses were performed using GraphPad Prism v.7 software. Results are expressed as percentage of the mean ± standard deviation (SD). Depending on the cases, data were analyzed either by 1-way analysis of variance (ANOVA) or Student’s *t*-test. A *p*-value < 0.05 was considered as statistically significant.

## 3. Results

### 3.1. The Synthesized Benzofuran-2-ones Have Antioxidant Capacity

Inspired by Nature’s employment of the catechol functional group, we realized the synthesis of new generation antioxidant compounds featured by a 3,3-disubstituted-3H-benzofuran-2-one scaffold bearing one or more hydroxyl groups on the aromatic ring (Table 1).

As previously reported, we accomplished the preparation of the desired phenolic derivatives **6**–**9** by an acid catalyzed domino reaction involving an initial Friedel-Crafts alkylation followed by intramolecular lactonization. Although compounds **6**–**8** presented a quite interesting antioxidant capacity evaluated using DPPH (rIC_50_ evaluated in both MeOH and ACN) and Cyclic Voltammetry (Ep measured in both H_2_O and ACN), the results presented in Table 1 highlight the remarkable antioxidant activity exhibited by the benzofuran-2-one **9** with rIC_50_ and Ep values comparable to and even better than those measured for Trolox. Considering the just depicted outcomes, we decided to carry out additional and more detailed *in vitro* tests to further endorse the promising antioxidant activity of the studied compounds as well as their toxicity.

### 3.2. The Benzofuran-2-one Derivatives ***6***–***9*** Show Low Toxicity in Undifferentiated SH-SY5Y Cells

**Figure 1:**
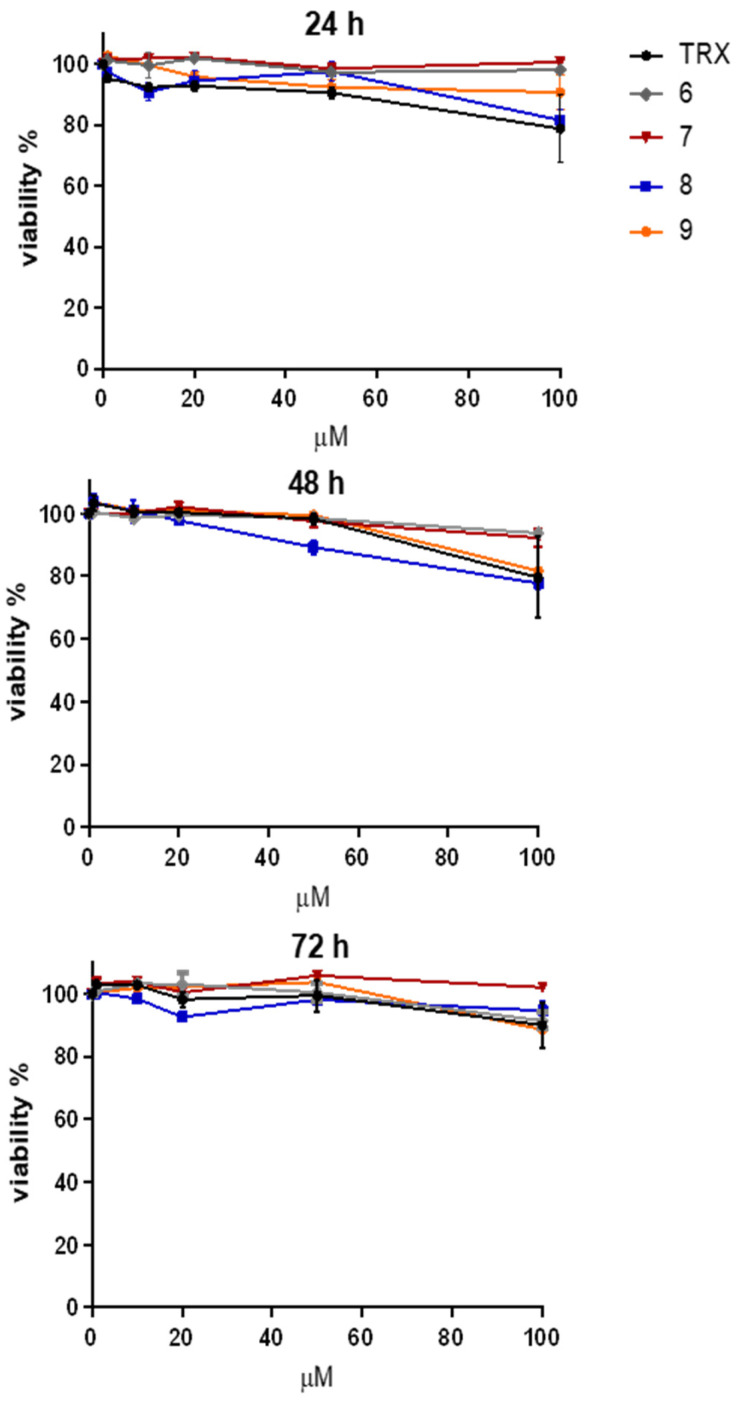
Effect of the benzofuran-2-ones **6**–**9** on the viability of undifferentiated SH-SY5Y cells. Trypan blue exclusion assay showing the time course (0–72 h) of the viability (expressed as percentage of control) of undifferentiated SH-SY5Y cells treated with increasing concentrations (0–100 μM) of the benzofuran-2-ones (**6**, **7**, **8** and **9**). Trolox (TRX) was included as a reference antioxidant.

The synthesized compounds **6**–**9** used for this study were already characterized for their antioxidant capacity by cyclic voltammetry and DPPH assay by our group [36]. Prior to test their efficacy *in vitro*, we investigated their cytotoxicity in undifferentiated SH-SY5Y cells. Figure 1 shows the time course (0–72 h) and dose–response curves (0–100 μM) of the viability of undifferentiated SH-SY5Y cells treated with the benzofuran-2-ones selected for our study (**6**, **7**, **8** and **9**). We also included Trolox (TRX) as a reference antioxidant. Since we did not observe any significant effect on the viability of our cellular model, we decided to adopt 10 μM as the working concentration for the compounds in the further experiments.

### 3.3. Catechol-Induced Intracellular ROS Levels and HO-1 Expression Affect the Proliferation of Undifferentiated SH-SY5Y Cells but Not Their Viability

**Figure 2:**
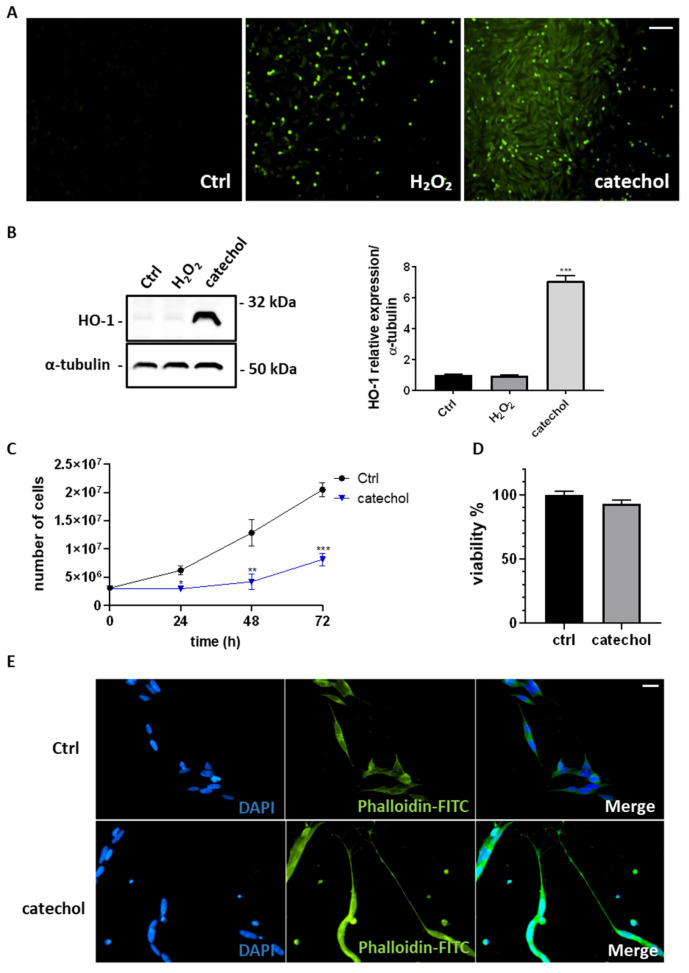
Effect of catechol stress on intracellular ROS production, HO-1 expression, proliferation, and viability of undifferentiated SH-SY5Y cells. (**A**) Representative fluorescence microphotographs showing intracellular ROS (in green) by dichlorodihydrofluorescein diacetate (DCF-DA) staining of undifferentiated SH-SY5Y cells exposed or not (Ctrl) for 6 h to 10 μM catechol compared to 250 μM H_2_O_2_. Magnification: 10×. Scale bar: 100 μM. (**B**) Western blot analysis showing HO-1 expression in undifferentiated SH-SY5Y cells exposed or not (Ctrl) for 6 h to 10 μM catechol compared to 250 μM H_2_O_2_. Results are expressed as the mean ± SD of three independent experiments. Statistical significance was assessed by one-way ANOVA (***: *p* < 0.001). (**C**) The graph shows the time course (0–72 h) of the proliferation (expressed as number of cells) of undifferentiated SH-SY5Y cells exposed or not (Ctrl, black line) to 10 μM catechol (blue line). Results are expressed as the mean ± SD of three independent experiments. Statistical significance was assessed by Student’s *t*-test (*: *p* < 0.05; **: *p* < 0.01; ***: *p* < 0.001). (**D**) Trypan blue exclusion assay showing the viability (expressed as percentage of control) of undifferentiated SH-SY5Y cells exposed or not (Ctrl) for 72 h to 10 μM catechol. (**E**) Fluorescence analysis showing the cytoskeleton (Phalloidin-FITC, in green), nuclei (DAPI, blue) and the merged channels (Merge) of undifferentiated SH-SY5Y cells exposed or not (Ctrl) for 72 h to 10 μM catechol. Magnification: 40×. Scale bar: 20 μM.

Catechol is a highly oxidant molecule resulting from the degradation of benzene [32]. Figure 2A shows the fluorescence analysis of DCF-DA staining highlighting how catechol is able to induce intracellular ROS in a greater extent than H_2_O_2_ in undifferentiated SH-SY5Y cells. We have also evaluated by the Western blot analysis the expression of HO-1 (Figure 2B), observing a 7-fold increase in undifferentiated SH-SY5Y cells treated with 10 μM catechol compared to 250 μM H_2_O_2_ and control cells. Furthermore, catechol slows the proliferation of undifferentiated SH-SY5Y cells (Figure 2C) with no effects on their viability (Figure 2D). Finally, fluorescence microscopy analysis performed by phalloidin-FITC incubation (Figure 2E) highlights that also the cytoskeleton was influenced by catechol stress with cells assuming a more fusiform morphology with prolonged neurites.

### 3.4. HO-1 Shows Perinuclear Localization upon Catechol Stress in Undifferentiated SH-SY5Y Cells

**Figure 3:**
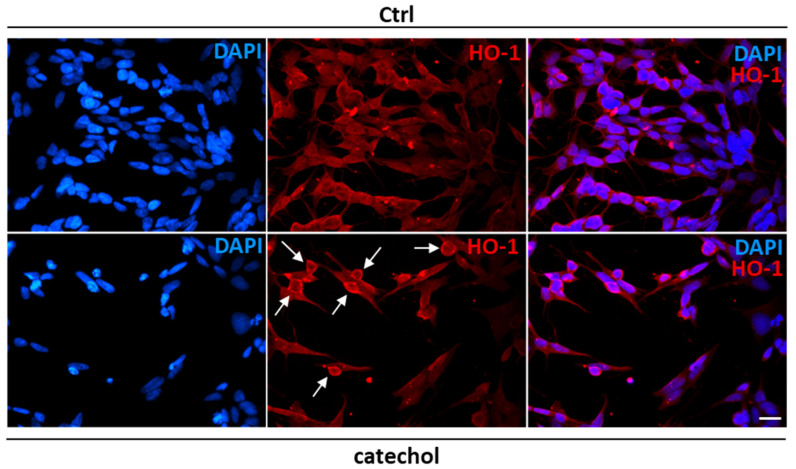
Heme oxygenase-1 localization after catechol stress in undifferentiated SH-SY5Y cells. Immunofluorescence analysis showing nuclei (DAPI, blue), HO-1 protein (red) and the merged channels in undifferentiated SH-SY5Y cells treated with 10 μM catechol for 6 h. White arrows indicate perinuclear localization of HO-1. Magnification: 40×. Scale bar: 20 μM.

The existence of a truncated form of HO-1 with nuclear localization generated after oxidative stress has been reported [41]. Here, we show by immunofluorescence analysis (Figure 3) that HO-1 localizes around the nucleus in most of the observed undifferentiated SH-SY5Y cells exposed to catechol stress compared to control cells where signals resulted more diffused within the cytoplasm.

### 3.5. Differentiated SH-SY5Y Cells Are More Sensitive to Catechol Stress Showing Lower HO-1 Induction than Undifferentiated Cells

**Figure 4:**
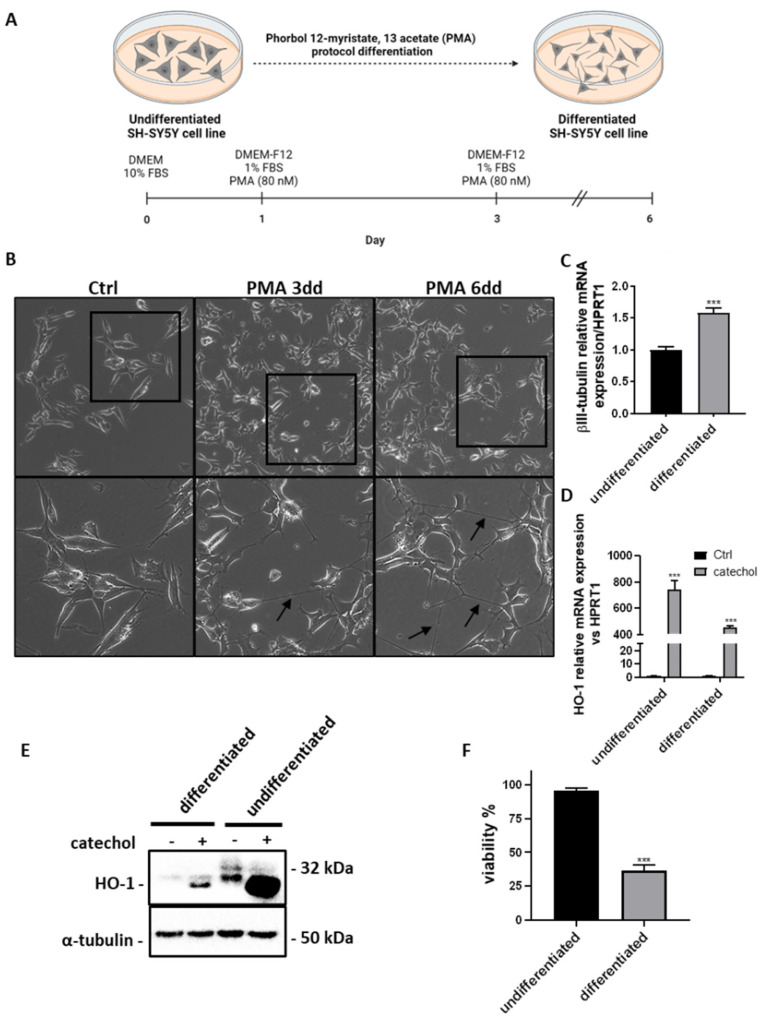
Effects of catechol stress on the expression of HO-1 and viability of differentiated and undifferentiated SH-SY5Y cells. (**A**) Schematic representation of SH-SY5Y cell differentiation protocol. The image was created with BioRender.com. (**B**) Microphotographs showing the time course (0–6 days) of SH-SY5Y cells differentiation by phorbol 12-myristate, 13 acetate (PMA). Black arrows indicate outgrowing neurites. Upper images: 10× magnification. Bottom images represent the magnification (20×) of the areas delimited by black squares in the upper images. (**C**) Real-time PCR analysis showing the relative mRNA expression levels of the neuronal differentiation marker βIII-tubulin in SH-SY5Y cells after 6 days of PMA differentiation (differentiated) compared to undifferentiated cells. Results are expressed as the mean ± SD of three independent experiments. Statistical significance was assessed by Student’s *t*-test (***: *p* < 0.001). (**D**) Real-time PCR analysis showing the relative mRNA expression levels of HO-1 in differentiated and undifferentiated SH-SY5Y cells exposed or not (Ctrl) to 10 μM catechol for 6 h. Results are expressed as the mean ± SD of three independent experiments. Statistical significance was assessed by Student’s *t*-test (***: *p* < 0.001). (**E**) Western blot analysis showing the presence and expression of HO-1 protein isoforms in differentiated and undifferentiated SH-SY5Y cells exposed or not to 10 μM catechol for 6 h. (**F**) Trypan blue exclusion assay showing the viability (expressed as percentage of control) of differentiated and undifferentiated SH-SY5Y cells exposed or not to 10 μM catechol for 24 h. Results are expressed as the mean ± SD of three independent experiments. Statistical significance was assessed by Student’s *t*-test (***: *p* < 0.001).

The differentiation of the SH-SY5Y neuroblastoma cell line is a well-established method for preliminary *in vitro* studies of neurodegenerative diseases [37]. Here, we show the differentiation protocol adopted for our studies (Figure 4A), which consists of exposing undifferentiated SH-SY5Y cells to 80 nM Phorbol 12-Myristate, 13 Acetate (PMA) in DMEM/F12 medium supplemented with 1% FBS for a minimum of 6 days. In Figure 4B, we highlight the characterization of our model of differentiation by observing the augmented neurites outgrow process after 3 and 6 days by optical microscopy analysis (Figure 4B). We also assessed the levels of the neuronal differentiation marker βIII-tubulin, which were higher by about 50% in differentiated cells compared to the undifferentiated counterpart (Figure 4C). Moreover, we exposed both cellular models to an oxidant agent as catechol, observing that undifferentiated cells show a higher induction of HO-1 both at the mRNA (Figure 4D) and protein (Figure 4E) level. Figure 4E also highlights the existence of three different isoforms of HO-1 with the 32 kDa full-length isoform being present only in undifferentiated SH-SY5Y cells. Furthermore, upon cell differentiation, SH-SY5Y cells express only a lower molecular weight isoform of HO-1, while its lightest isoform appears only under catechol exposure regardless of differentiation. Finally, we evaluated the viability of SH-SY5Y cells exposed to catechol and observed that only differentiated cells are sensitive to oxidative stress, with high cell mortality percentages reaching about 70% after 24 h (Figure 4F).

### 3.6. The Benzofuran-2-ones ***6***–***9*** Reduce Catechol-Induced Intracellular ROS Production in Differentiated SH-SY5Y Cells

**Figure 5:**
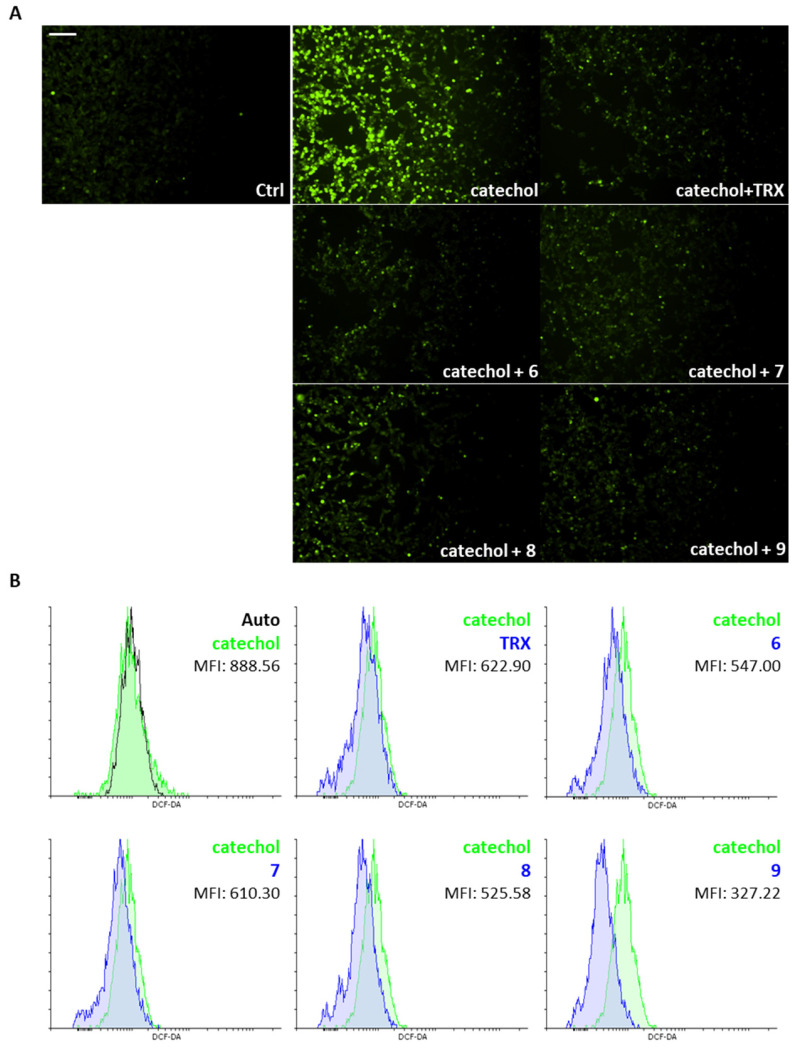
Effects of the benzofuran-2-ones **6**–**9** on catechol-induced intracellular ROS production in differentiated SH-SY5Y cells. (**A**) Representative fluorescence microphotographs showing intracellular ROS (in green) by dichlorodihydrofluorescein diacetate (DCF-DA) staining of differentiated SH-SY5Y cells exposed or not (Ctrl) for 6 h to 10 μM catechol with or without 10 μM benzofuran-2-ones (**6**, **7**, **8** and **9**). Trolox (TRX, 10 μM) was included as a reference antioxidant. Magnification: 10×. Scale bar: 100 μM. (**B**) Cytofluorimetric analysis of dichlorodihydrofluorescein diacetate (DCF-DA) staining showing intracellular ROS levels, expressed as Mean Fluorescence Intensity (MFI), of differentiated SH-SY5Y cells exposed or not for 6 h to 10 μM catechol (in green) with or without 10 μM benzofuran-2-ones (**6**, **7**, **8** and **9**, in blue). Trolox (TRX, 10 μM, in blue) was included as a reference antioxidant.

The evaluation of the antioxidant capacity of the novel benzofuran-2-ones was performed in differentiated SH-SY5Y cells exposed to catechol stress. In Figure 5A, we show that all the newly synthesized molecules are able to significantly reduce the levels of intracellular ROS as qualitatively assessed by the fluorescence analysis of DCF-DA staining. In particular, we observe strong signals when cells are exposed for 6 h to 10 μM catechol, which are prevented by the concomitant treatment with compounds **6**, **7**, **8**, **9** or the reference antioxidant, Trolox. Further, we quantified the lowering of intracellular ROS levels for all the compounds by the cytofluorimetric analysis of DCF-DA staining. Figure 5B highlights the extraordinary potential of compound **9** to reduce intracellular ROS levels as denoted by the lowest Mean Fluorescence Intensity values (MFI: 327.22 for **9** *vs*. 888.56 for catechol and 622.9 for TRX).

### 3.7. Compound ***9*** Reduces Catechol-Induced Nuclear Fragmentation While Causing the Accumulation of HO-1 in the Perinucleus of Damaged Differentiated SH-SY5Y Cells

**Figure 6:**
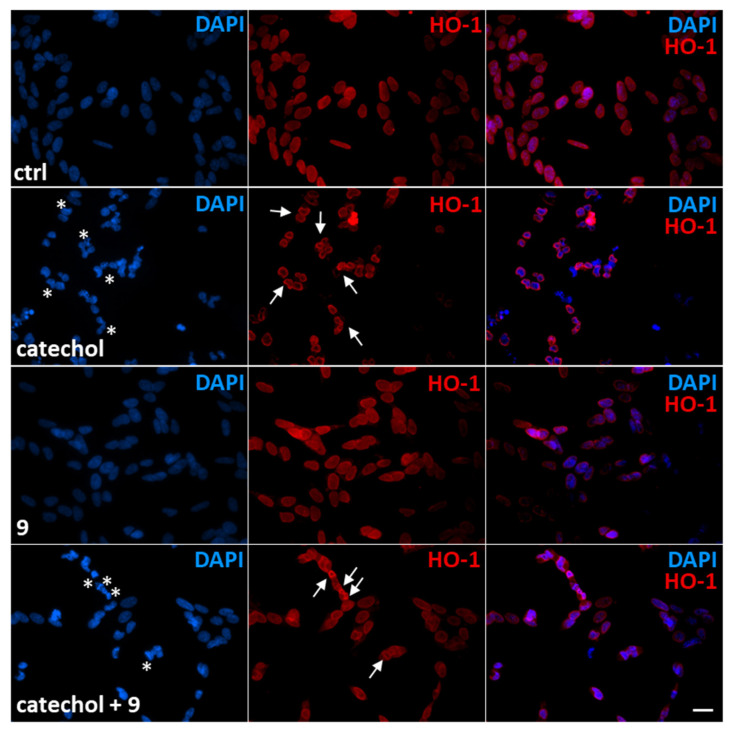
Effects of the compound **9** on nuclear fragmentation and HO-1 localization in differentiated SH-SY5Y cells exposed to catechol. Immunofluorescence analysis showing nuclei (DAPI, blue), HO-1 protein (red) and the merged channels in differentiated SH-SY5Y cells treated or not (ctrl) with 10 μM catechol for 6 h in presence or not of 10 μM compound **9**. White asterisks indicate damaged nuclei. White arrows indicate perinuclear localization of HO-1. Magnification: 40×. Scale bar: 20 μM.

Intracellular ROS production resulting from cellular exposure to oxidants leads to DNA damage and nuclear fragmentation [42]. In Figure 6, we observed that in differentiated SH-SY5Y control cells HO-1 is weakly expressed and mainly localized within the nucleus. When we expose these cells to catechol for 6 h, we assist nuclear fragmentation (evaluated by DAPI staining of cell nuclei) accompanied by a perinuclear translocation of HO-1. The effect of the molecule **9** on catechol-treated differentiated SH-SY5Y cells strongly reduces the amount of DNA-damaged cells, which are also characterized by high-intensity signals for HO-1 in the perinuclear region. Finally, we do not observe any significant changes in HO-1 expression and localization when the compound **9** alone is added to differentiated SH-SY5Y cells compared to control cells.

### 3.8. Compound ***9*** Boosts Catechol-Induced HO-1 Expression Protecting Differentiated SH-SY5Y Cells from Cell Death

**Figure 7:**
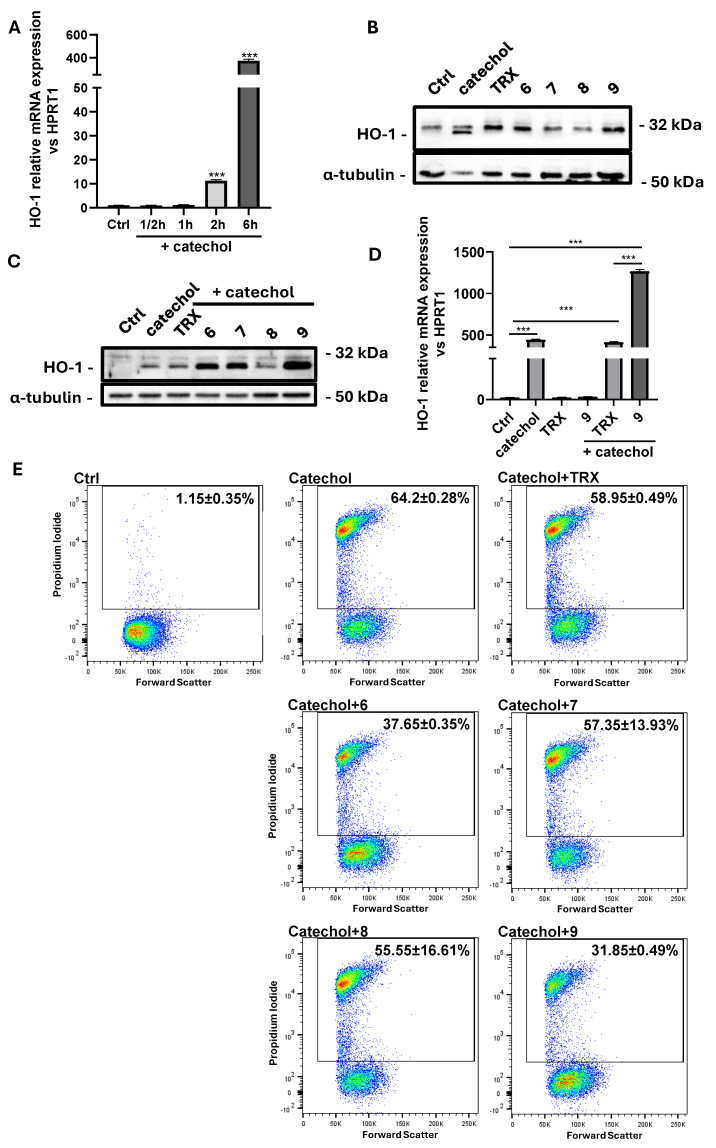
Effects of the benzofuran-2-ones **6**–**9** on HO-1 expression and viability of differentiated SH-SY5Y cells exposed to catechol stress. (**A**) Real-time PCR analysis showing the time course (0–6 h) of induction of HO-1 mRNA expression under catechol stress in differentiated SH-SY5Y cells. Results are expressed as the mean ± SD of three independent experiments. Statistical significance was assessed by one-way ANOVA (***: *p* < 0.001). (**B**) Western blot analysis showing the expression of HO-1 protein in differentiated SH-SY5Y exposed or not (Ctrl) for 6 h to 10 μM catechol or to 10 μM benzofuran-2-ones (**6**, **7**, **8** and **9**). Trolox (TRX) was included as a reference antioxidant. (**C**) Western blot analysis showing the expression levels of HO-1 protein in differentiated SH-SY5Y exposed or not (Ctrl) for 6 h to 10 μM catechol in presence or not of 10 μM benzofuran-2-ones (**6**, **7**, **8** and **9**). Trolox (TRX) was included as a reference antioxidant. Results are expressed as the mean ± SD of three independent experiments. (**D**) Real-time PCR analysis showing HO-1 mRNA levels in differentiated SH-SY5Y cells exposed or not (Ctrl) for 6 h to 10 μM catechol and to 10 μM TRX or **9** in presence or not of 10 μM catechol. Results are expressed as the mean ± SD of three independent experiments. Statistical significance was assessed by one-way ANOVA (***: *p* < 0.001). (**E**) Cytofluorimetric analysis of propidium iodide (PI) staining showing necrotic differentiated SH-SY5Y cells (expressed as percentage) exposed or not (Ctrl) for 24 h to 10 μM catechol in presence or not of 10 μM benzofuran-2-ones (**6**, **7**, **8** and **9**). Trolox (TRX) was included as a reference antioxidant.

HO-1 is an oxidative stress-inducible enzyme with well-known neuroprotective properties [43]. In Figure 7, we show the effects of the newly synthesized benzofuran-2-ones on catechol induced HO-1 expression and cell death. In detail, we performed a time-course by analyzing the levels of HO-1 transcripts in differentiated SH-SY5Y cells exposed to 10 μM catechol (Figure 7A), observing an extraordinary peak of induction after 6 h (about 400-folds compared to control cells, *p* < 0.001), which begins to be appreciable already after 2 h of treatment (about 10-folds compared to control cells, *p* < 0.001). Catechol stress is also able to induce the expression of the lower molecular weight isoform of HO-1 protein, while the antioxidants alone do not show any significant effects on the expression of this enzyme (Figure 7B). We also evaluated the ability of the benzofuran-2-ones to influence catechol-induced HO-1 expression. Figure 7C highlights that molecules **6**, **7** and **9** have the ability to induce HO-1 levels under catechol-induced oxidative stress, with the compound **9** showing the greatest effects. In the same way, **9** shows the same effects also on the induction of HO-1 mRNA when differentiated SH-SY5Y cells are exposed to catechol stress (Figure 7D). Finally, we evaluated the effects of the newly synthesized antioxidants on limiting cell death after the exposure to oxidative stress by cytofluorimetric analysis of PI staining. Figure 7E highlights the great capacity of **9** in protecting differentiated SH-SY5Y cells from catechol-induced cell death (dead cells: 31.85 ± 0.49% for **9** *vs.* 64.2 ± 0.28% for catechol). The compound **6** also shows interesting effects in preventing cell death under oxidative stress conditions (dead cells: 37.65 ± 0.35%).

## 4. Discussion

The main findings reported in this study show that (i) all of the selected benzofuran-2-ones **6**–**9** showed low toxicity in the *in vitro* neuronal model investigated; (ii) the tested compounds acted as antioxidants being able to reduce catechol-induced intracellular ROS levels in differentiated SH-SY5Y cells; (iii) differentiated SH-SY5Y cells are more sensitive to catechol stress being less able to upregulate HO-1 compared to the undifferentiated counterpart; (iv) in differentiated and undifferentiated SH-SY5Y cells catechol stress causes the upregulation of a lower-molecular-weight HO-1 isoform with perinuclear localization; (v) compound **9** shows the greatest antioxidant capacity in terms of intracellular ROS level reduction, HO-1 level induction, and viability preservation of differentiated SH-SY5Y cells exposed to catechol stress.

Neurodegeneration associated with age is a condition characterized by the slow and progressive loss of neuronal cells and/or their functionality [44,45]. Beyond familial predisposition [46], many environmental factors contribute to accelerate this pathological aging process. Pollutants [47], smoke [48], alcohol [49], non-healthy and sedentary lifestyle [50], are considered main risk factors for degenerative diseases of the CNS as well as the heart and other organs. One of the main stressor determinants for neurodegenerative diseases is represented by OS whose balance is considered the holy grail for health and longevity [51]. For this reason, both natural bioactive compounds as well as researching on the synthesis of more stable, potent, high-yielded, and cost-effective molecules have always attracted interests to counteract the pressure of ROS on cellular organelles and biological macromolecules [52].

To this regard, in recent years our group focused on novel and alternative routes to synthesize a new generation of promising antioxidant compounds via domino Friedel-Crafts/lactonization reaction [36]. In these molecules, the 3*H*-benzofuran-2-one core is disubstituted at the C3 position and decorated with one or more OH groups on the aromatic moiety. Their antioxidant capacity has already been assessed by both DPPH assay and cyclic voltammetry [36]. Considering our observations, we selected four compounds for *in vitro* testing in a neuronal model exposed to OS, a chronically occurring event of the neurodegenerative process.

We performed our experiments on SH-SY5Y cells upon a differentiation process induced by PMA (Figure 4). The SH-SY5Y cell line represents an undifferentiated neuroblastoma whose differentiation can be obtained by several inducers, including retinoic acid, and PMA [53]. This method is widely accepted by the literature and allows a genetic switch and morphological changes to mimic the normal neuronal context most likely [54]. Some studies establish that the higher degree of differentiation is reached by treating SH-SY5Y cells with both retinoic acid and PMA [55]. In this study, we reported a good differentiation for SH-SY5Y cells by only PMA administration for at least 6 days, highlighted by the upregulation of the neuronal differentiation marker βIII-tubulin mRNA levels and an appreciable outgrowth of neurites. The degree of differentiation of SH-SY5Y cells that we obtain by the adopted protocol is sufficient for our aims. In fact, differentiated cells clearly show higher sensitivity to oxidative stress compared to undifferentiated cells. This is totally in line with many other observations [56,57,58], even if some controversial studies described opposite effects [59].

In this study, we opted to induce oxidative stress in our cells by catechol treatment. Catechol (1,2-dihydroxybenzene) is a metabolite of the bioactivation of benzene with well-known genotoxic, immunotoxic, and haematotoxic properties [60,61,62]. Benzene is present as a volatile and environmental contaminant compound and represents a toxicity risk for long-term exposed workers. Hazardous metabolites of benzene are hydroquinone, phenol, and catechol [63]. Catechol exerts its toxicity by undergoing auto-oxidation in aqueous solution, at physiological pH, forming quinones and semiquinone radicals, both with a greater reactivity compared to catechol. Besides industrial chemicals, catechol is also present in foods and cigarettes smoke. Catechol itself is not responsible for the damage to biological macromolecules, but it was shown that protein and DNA damage is due to catechol-induced ROS generation by redox reactions [31].

In this study, we clearly showed that catechol-induced oxidative stress in SH-SY5Y cells is qualitatively and quantitatively higher than that induced by H_2_O_2_. This is evidenced by our results showing elevated intracellular ROS levels in catechol-treated SH-SY5Y cells compared to H_2_O_2_ and control cells (Figure 2 and Figure 5). Adding H_2_O_2_ is a widely used method to trigger oxidative stress in cell cultures [64]. The main mechanism for oxidative damage is thought to be the Fenton’s reaction between H_2_O_2_ and Fe^2+^ ions generating the OH radical with high reactivity properties [65]. In cells, the oxidative stressor generators are represented by mitochondria, where the oxygen-consuming cellular respiration plays a pivotal role in cell damage [66]. However, the treatment with exogenous H_2_O_2_ does not always recapitulate the mechanism underlying the endogenous oxidative damage. The H_2_O_2_ issue arises when the adopted concentrations become supraphysiological [67]. Further, recurring to high doses of H_2_O_2_ surely are required for appreciable deleterious effects on cells (above 100 μM), but this is far away from mimicking the production of endogenous H_2_O_2_, which should be physiologically compartmentalized [68]. All these considerations may explain our observation that only catechol, contrarily to H_2_O_2_, was able to strongly induce both HO-1 protein and mRNA levels.

HO-1 is a 32-kDa heat-shock protein whose upregulation is a well-established mechanism of oxidative stress response as well as cell adaptation to stress [69]. HO-1 plays crucial roles in CNS in terms of neuroprotection. It has been demonstrated that HO-1 overexpression in neuroblastoma cells protects them from oxidative stress induced by β-amyloid and H_2_O_2_ [19]. Similar observations were highlighted by recurring to natural compounds as tetrahydroxystilbene glucoside, which acts as a neuroprotective agent by stimulating HO-1 expression [70]. Further, animal models overexpressing HO-1 in neurons show higher protection after cerebral ischemia by mechanisms lowering lipid peroxidation and upregulating the Bcl-2 anti-apoptotic protein [71]. However, high HO-1 expression has been also associated with neurodegeneration and neuronal damage. In particular, high levels of HO-1 are described in AD brains [72]. HO-1 is also overexpressed in dopaminergic neurons constituting Lewy bodies in PD [73]. In normal aging, HO-1 can be induced in specific subpopulations of neurons as an adaptive defense mechanism to oxidative stress [74]. In this context, we have demonstrated that HO-1 is overexpressed in the aging mouse cortex as a consequence of oxidative stress and, regulating oxysterols production, exacerbates this condition under the control of the early gene Egr-1 [22]. However, the role of HO-1 is strictly related to the intensity of the insult, duration, and the signaling pathways activated for HO-1 stimulation, which can result in cytotoxicity or cytoprotection depending on the involvement of Nrf2 [74]. Also, the differentiation status of neuronal cultures can influence the response to oxidative stress. To this regard, here we report that the antioxidant defense is different among differentiated and undifferentiated SH-SY5Y cells, with the latter being the more resistant to oxidative stress (Figure 4F). This could be explained by the fact that undifferentiated cells possess the machinery to strongly upregulate the protective HO-1 enzyme. Consequently, undifferentiated SH-SY5Y cells respond to catechol-induced intracellular ROS levels by upregulating HO-1 at high levels, leading to cell proliferation slowing, cytoskeletal rearrangements, and cell survival. However, when SH-SY5Y cells undergo the process of differentiation, we assist to a less capacity to induce HO-1 levels under oxidative stress, which does not permit to cells to resist to the oxidative insult (Figure 4E,F). This is in line with the study conducted by Piras and colleagues showing that differentiation of SH-SY5Y cells by ATRA (all-trans retinoic acid) impairs the induction of HO-1 via Bach1, which results in an increased sensitivity to H_2_O_2_-induced oxidative stress [58].

In light of these considerations, we decided to test the novel antioxidant compounds and their ability to counteract catechol-induced oxidative stress and protect differentiated SH-SY5Y cells from cell death. Firstly, we qualitatively show that all the compounds tested are able to reduce intracellular ROS levels assessed by fluorescent live microscopy of DCF-DA staining in differentiated SH-SY5Y cells (Figure 5A). Our results are comparable to cells treated with a reference antioxidant, the hydro soluble analog of vitamin E, Trolox (TRX). We have also quantified this reduction by cytofluorimetric analysis of these cells incubated with the DCF-DA fluorescent probe and observed how compound **9** above all acts as an antioxidant of extraordinary capacity, reducing intracellular ROS levels 2-fold more than TRX (Figure 5B).

Possibly, compounds **6** and **7**, due to the presence of an OH in *para* position with respect to the oxygen in the five-membered ring, may react with ROS to generate a phenoxyl radical, which, similarly to Trolox, is able to delocalize an electron around the aromatic ring. Compound **9** is characterized by a catechol group whose well-known antioxidant activity is further enhanced by the synergistic action of one of the two hydroxyl groups which, being in the *ortho* position, should easily delocalize an electron and stabilize the corresponding radical generated by ROS. Such hypothesis is further supported by the low antioxidant capacity exhibited by compound **8**, where the hydroxyl group in *meta* position is not able to delocalize any electron.

Differentiated SH-SY5Y cells show sensitivity to catechol and show a peak of induction of the HO-1 transcript (Figure 7A) and protein (Figure S1) at 6 h. After this time, we can appreciate the induction of a lower-molecular-weight protein isoform by Western blot analysis (Figure 7B and Figure S1). Some studies have described the expression of a truncated, 28 kDa isoform of HO-1 upon oxidative stress with a nuclear localization. The proposed underlying mechanism is that when a cell is exposed to oxidants, this lighter isoform of HO-1 is induced, enters the nucleus, and activates genes involved in the antioxidant response [75].

Surprisingly, when we compared HO-1 protein expression in catechol-treated differentiated and undifferentiated SH-SY5Y cells, we observed that HO-1 protein is present in three different isoforms, which vary along cell differentiation and whether cells are exposed to oxidative stress (Figure 4E). In particular, by matching our results with the immunofluorescence analysis of HO-1 (Figure 3 and Figure 6), we observed that the full length 32-kDa isoform has a cytoplasmic localization. Further, upon cell differentiation we assisted the enrichment of a nuclear isoform with a lower molecular weight that could correspond to the already described truncated 28-kDa isoform [41,75]. Interestingly, when both cellular models are treated with catechol, a lighter HO-1 isoform is induced and localizes to the perinucleus. Therefore, understanding the mechanism by which HO-1 is processed and mobilizes within cell compartments in response to various conditions becomes of extreme importance and surely needs further investigation. The perinuclear translocation of HO-1 under oxidative stress that we observed in this study has been also described by Collinson et al. in yeast cells treated with H_2_O_2_ [32].

Of note, we did not observe this lowest-molecular-weight HO-1 isoform either in control cells or when cells were treated with the antioxidants alone (Figure 7B). However, when we added the antioxidants to catechol-stressed differentiated SH-SY5Y cells, we assisted the upregulation of the above-described isoform, exclusively with molecules **6**, **7** and **9** (Figure 7C). Again, compound **9** was the molecule upregulating the most HO-1 protein isoform and mRNA levels.

The precise molecular mechanisms underlying HO-1 induction, processing and mobilization under oxidative stress exerted by the selected benzofuran-2-ones need further investigation and go beyond the aim of the present work which was the evaluation of the efficacy of newly synthesized molecules with potential antioxidant activity in a cellular model of neurodegeneration.

We believe that the localization of HO-1 is crucial for the response to oxidative stress. To this purpose, here we also describe the great ability of compound **9** to reduce catechol-induced nuclear fragmentation while causing the accumulation of HO-1 in the perinucleus of damaged differentiated SH-SY5Y cells. This observation suggests a possible role for HO-1 in DNA repair in stressed neuronal cells, a mechanism described by Otterbein et al. who highlighted the modulation of DNA repair by HO-1 and CO through ataxia-telangiectasia mutated protein (ATM) in various tissues, brain excluded [76].

As already mentioned, HO-1 is a double-edge-sword enzyme because it shows protective and cytotoxic effects depending on its levels of induction [77]. In this context, the strong induction of HO-1 reflects its neuroprotective properties by greatly reducing catechol-induced cell death. Moreover, another promising molecule to be monitored is **6**, which shows good HO-1 inducing properties as well as good protective abilities.

As previously depicted, compounds **6** and **9** are quite similar in terms of molecular structure, nevertheless the very small differences could contribute to explain the better antioxidant properties of **9**. Specifically, compound **6** has a single hydroxyl group in *para* position with respect the oxygen in the five membered ring, where compound **9** has the OH group in *ortho* position. Such group together with the second OH moiety should be involved not only in the enhanced antioxidant activity but could also affect HO-1 expression. Likewise, the influence of the CF_3_ moiety instead of the more reactive CO_2_Et could not be excluded. To this regard, detailed investigations to validate our hypothesis and better elucidate the action mechanism of the benzofuran-2-ones as well as structure activity relationships (SAR) studies are ongoing in our laboratories and will be reported in due course.

In conclusion, the ability to induce HO-1 is the goal of many pharmacological interventions [78,79]. Upregulating HO-1 expression has shown good protective roles for many pathologies, neurodegenerative diseases included [80,81,82,83]. Our molecules do not act as HO-1 inducers by themselves. However, especially compound **9** in a greater extent can boost HO-1 expression in the precise time and place the cell needs it the most for its survival. In fact, the great ability of this molecule to induce HO-1 under catechol stress translates in a great neuroprotective ability, opening the way to consider benzofuran-2-ones as novel and effective antioxidant tools in controlling oxidative stress. This may represent an adjuvant promising strategy for many neurodegenerative disorders.

## Figures and Tables

**Table 1:**
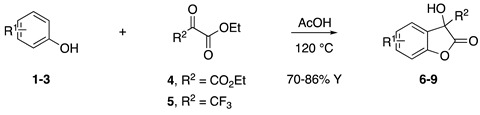
Synthesized 3,3-disubstituted-3*H*-benzofuran-2-one **6**–**9** and their antioxidant activity evaluated using DPPH assay and Cyclic Voltammetry.

Entry	Substrate		R^2^	Product		rIC_50_ ^1^	rIC_50_ ^2^	E_p_^ox^ (V) ^3^	E_p_^ox^ (V) ^4^
a	**1**	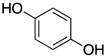	CO_2_Et	**6**	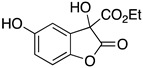	0.31	4.26	0.72	1.62
b	**1**	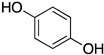	CF_3_	**7**	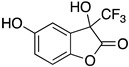	0.22	1.69	0.62	1.78
c	**2**	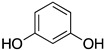	CF_3_	**8**	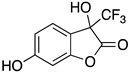	3.52	4.47	1.01	1.92
d	**3**	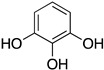	CF_3_	**9**	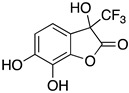	0.18	0.17	0.85	1.81
e	**TRX**	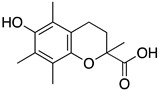				0.23	0.22	0.52	1.08

^1^ Antioxidant capacity towards DPPH· in methanol (MeOH), ^2^ Antioxidant capacity towards DPPH· in acetonitrile (ACN); ^3^ First oxidation peaks (E_p_^ox^) from CV in aqueous medium; ^4^ First oxidation peaks (E_p_^ox^) from CV in ACN. All the peak potetials are referred to SCE (Saturated Calomel Electrode, SCE).

## Data Availability

Main data generated or analyzed in this study are included in this article. Details are available from the corresponding author on reasonable request.

## Supplementary Materials

The following supporting information can be downloaded at: https://www.mdpi.com/article/10.3390/life14040422/s1, Figure S1: effects of compound **9** on HO-1 expression of differentiated SH-SY5Y cells exposed to catechol stress. Figure S2: dose–response curves and dose-escalation evaluation of the effects of synthetic benzofuran-2-ones on the viability of differentiated SH-SY5Y cells exposed or not to catechol stress. Figure S3: clarification on HO-1 isoforms presented in Figure 4E. Figure S4: additional oil-immersion 60× magnification immunofluorescence analysis images of Figure 6.
