# Supplementary figures and images for: In Vitro Evaluation of the Antioxidant Capacity of 3,3-Disubstituted-3H-benzofuran-2-one Derivatives in a Cellular Model of Neurodegeneration
Source: Life (Basel). 2024 Mar 22;14(4):422. doi: 10.3390/life14040422 (PMC11051253; doi:10.3390/life14040422)

Figure S3

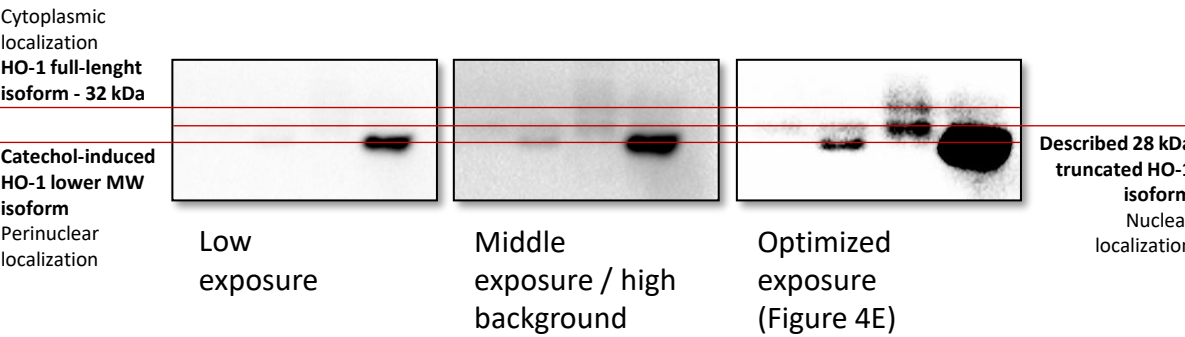

Supplement: Supplementary file 1 [file life-14-00422-s001.zip › Figure S3_new.pdf]

Figure S4

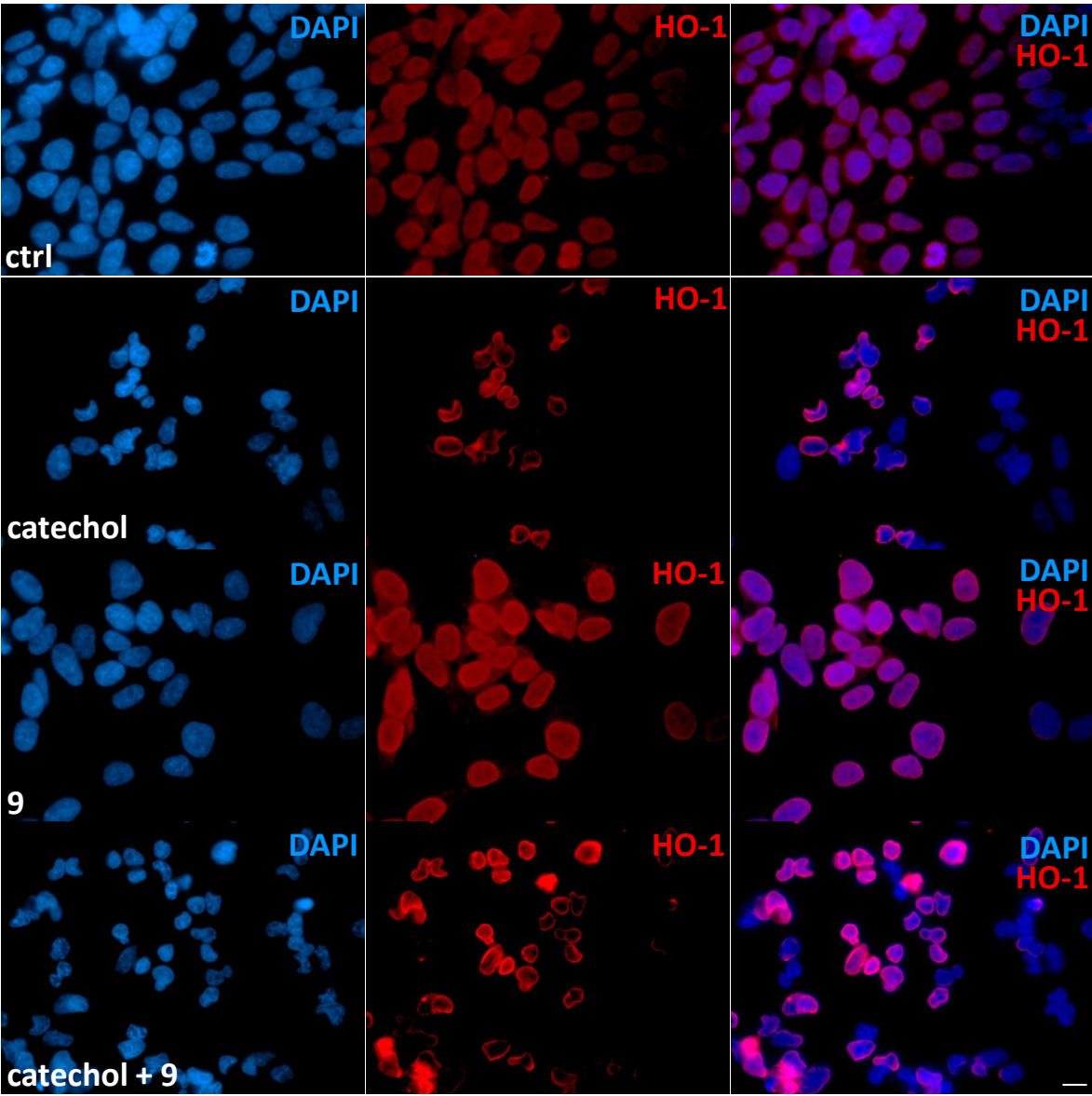

Supplement: Supplementary file 1 [file life-14-00422-s001.zip › Figure S4_new.pdf]

Figure S1

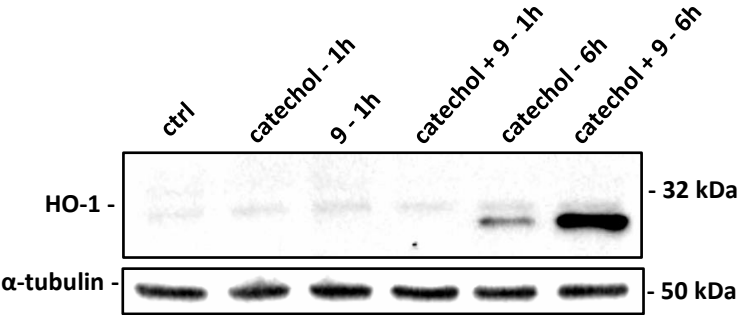

Supplement: Supplementary file 1 [file life-14-00422-s001.zip › Figure S1_new.pdf]

Figure S2

**A**

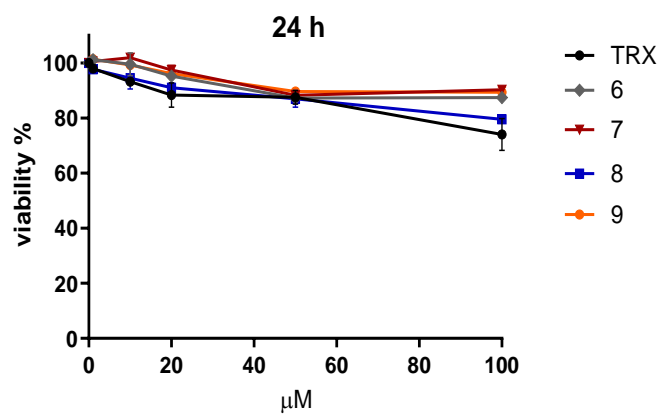

**B**

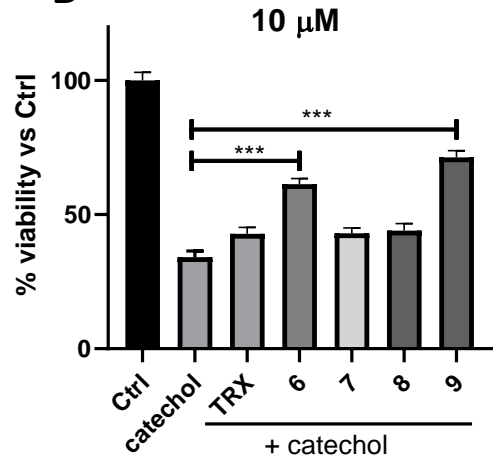

**C**

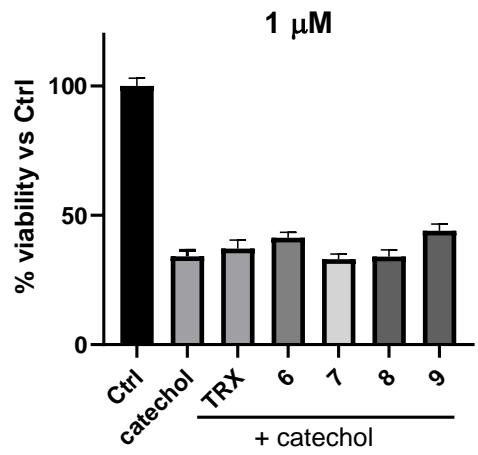

Supplement: Supplementary file 1 [file life-14-00422-s001.zip › Figure S2_new.pdf]
